# Characterization of Zinc Influx Transporters (ZIPs) in Pancreatic β Cells: ROLES IN REGULATING CYTOSOLIC ZINC HOMEOSTASIS AND INSULIN SECRETION*

**DOI:** 10.1074/jbc.M115.640524

**Published:** 2015-05-12

**Authors:** Ying Liu, Battsetseg Batchuluun, Louisa Ho, Dan Zhu, Kacey J. Prentice, Alpana Bhattacharjee, Ming Zhang, Farzaneh Pourasgari, Alexandre B. Hardy, Kathryn M. Taylor, Herbert Gaisano, Feihan F. Dai, Michael B. Wheeler

**Affiliations:** From the ‡ Department of Physiology, University of Toronto, Toronto, Ontario M5S 1A8, Canada and; the § Breast Cancer Molecular Pharmacology Unit, School of Pharmacy and Pharmaceutical Sciences, Cardiff University, Redwood Building, King Edward VIIth Avenue, Cardiff CF10 3NB United Kingdom

**Keywords:** exocytosis, glucose, insulin secretion, pancreatic islet, zinc, GLP1, insulin granule, zinc influx transporter

## Abstract

Zinc plays an essential role in the regulation of pancreatic β cell function, affecting important processes including insulin biosynthesis, glucose-stimulated insulin secretion, and cell viability. Mutations in the zinc efflux transport protein ZnT8 have been linked with both type 1 and type 2 diabetes, further supporting an important role for zinc in glucose homeostasis. However, very little is known about how cytosolic zinc is controlled by zinc influx transporters (ZIPs). In this study, we examined the β cell and islet ZIP transcriptome and show consistent high expression of ZIP6 (Slc39a6) and ZIP7 (Slc39a7) genes across human and mouse islets and MIN6 β cells. Modulation of ZIP6 and ZIP7 expression significantly altered cytosolic zinc influx in pancreatic β cells, indicating an important role for ZIP6 and ZIP7 in regulating cellular zinc homeostasis. Functionally, this dysregulated cytosolic zinc homeostasis led to impaired insulin secretion. In parallel studies, we identified both ZIP6 and ZIP7 as potential interacting proteins with GLP-1R by a membrane yeast two-hybrid assay. Knock-down of ZIP6 but not ZIP7 in MIN6 β cells impaired the protective effects of GLP-1 on fatty acid-induced cell apoptosis, possibly via reduced activation of the p-ERK pathway. Therefore, our data suggest that ZIP6 and ZIP7 function as two important zinc influx transporters to regulate cytosolic zinc concentrations and insulin secretion in β cells. In particular, ZIP6 is also capable of directly interacting with GLP-1R to facilitate the protective effect of GLP-1 on β cell survival.

## Teaser

**Background:** Zinc influx transporters (ZIPs), together with zinc efflux transporters (ZnTs), regulate cellular zinc homeostasis.

**Results:** Down-regulation of ZIP6 and ZIP7 expression impairs glucose-stimulated insulin secretion via reduced first-phase insulin exocytosis.

**Conclusion:** ZIP6 and ZIP7 are functionally important for maintaining proper insulin secretion in pancreatic β cells.

**Significance:** ZIP6 and ZIP7 represent novel proteins that contribute to the insulin secretory pathway.

## Introduction

Zinc is a critical micronutrient required for numerous cellular processes, including DNA and protein synthesis, enzyme activity, and intracellular signaling (1, 2). Importantly, zinc plays an essential role in insulin-producing pancreatic islet β cells, in which zinc content is among the highest in the body (3). Intracellular zinc homeostasis is tightly regulated by two major families of zinc transport proteins, the Zrt- and Irt-like proteins (ZIPs)^7^ and zinc efflux (ZnTs) transporters. In general, the ZIP family is responsible for zinc influx into the cytosol from extracellular sources and intracellular organelles, and, conversely, the ZnT family is responsible for efflux. The fine balance between specific ZIPs and ZnTs regulates both cytosolic and intraorganelle zinc concentrations.

At present, 10 members of the ZnT family (ZnT1–10, encoded by Slc30a1–10) and 14 members of the ZIP family (ZIP1–14, encoded by Slc39a1–14) have been identified in humans. The expression of specific ZIPs and ZnTs in cells is tissue-dependent and closely tied to cellular function (4, 5). Previous work by our group and others using whole body and pancreatic β cell-specific ZnT8 knockout mouse models has highlighted the importance of zinc and zinc transporters in β cell function and glucose homeostasis (6–9). These studies showed, among other things, that ZnT8 is required for proper insulin biosynthesis and crystallization, presumably by regulating zinc entry into insulin secretory vesicles (10, 11). On the basis of these findings, it is not surprising that studies in humans point to a role for ZnT8 in the pathophysiology of type 2 diabetes (7, 12, 13), further exemplifying the importance of zinc transporters in β cells.

Beyond Znt8, however, little is known about how zinc levels are regulated in β cells. Most importantly, it is not well understood how zinc enters β cells and how this entrance is regulated to affect cytosolic zinc concentrations. We have shown previously that voltage-gated calcium channels (VGCCs) can act as zinc influx transporters (14) during cellular membrane depolarization associated with high glucose concentrations. However, zinc also enters the β cell under resting/low-glucose conditions, indicating that there is another mechanism of influx that is likely independent of VGCCs. Our previous work has identified several ZIPs in β cells that may contribute to zinc homeostasis. Currently, limited information is available regarding the function of these ZIPs in pancreatic β cells, although a few studies have suggested that ZIPs, specifically ZIP4 (15), may play some role in the regulation of pancreatic β cell zinc homeostasis (16, 17).

In this study, we show consistent high expression of ZIP6 and ZIP7 across islets and β cell lines, suggesting that they are prominent zinc influx transporters in pancreatic β cells. Our data suggest that ZIP6 and ZIP7 work together with the ZnT family to regulate cytosolic zinc homeostasis in this cell type. Functionally, down-regulation of ZIP6 and ZIP7 in pancreatic β cells reduces cytosolic zinc content, which causes impaired insulin exocytosis and, therefore, reduced glucose-stimulated insulin secretion (GSIS). Interestingly, we also show that ZIP6 interacts physically with the glucagon-like peptide 1 receptor (GLP-1R). This interaction mediates the protective effects of GLP-1 on β cell apoptosis.

## Materials and Methods

### 

#### 

##### Cell Culture and Transfection

MIN6 β cells were cultured in DMEM (4500 mg/l glucose and l-glutamine; Sigma) supplemented with 10% fetal bovine serum, 1% penicillin/streptomycin, and 50 μm β-mercaptoethanol. INS-1 832/3 cells (from Dr. Chris Newgard at Duke University, Durham, NC) were maintained in RPMI 1640 medium (11.1 mm d-glucose) supplemented with 10% FBS, 1% penicillin/streptomycin 10 mm HEPES, 2 mm l-glutamine, 1 mm sodium pyruvate, and 50 μm β-mercaptoethanol. Cells were incubated at 37 °C in a 5% CO_2_ humidified incubator. The culture medium was changed every 48–72 h until the optimal confluence for treatment. MIN6 β cells were cultured in 96-well plates until ∼80% confluent. Cells were transfected with 30 pmol/well of targeted siRNA specific to ZIP6 and ZIP7 or a non-silencing control siRNA (scramble) mixed with Lipofectamine RNAiMAX (Life Technologies) transfection reagent according to the instructions of the manufacturer. Alternatively, cells were transfected with 0.25 μg/well plasmid cDNA targeted specifically to overexpress ZIP6 (pcDNA3.1-ZIP6-HA, a gift from Kyle W. Sloop, Lilly Research Laboratories) and ZIP7 (pcDNA3.1-ZIP7-FLAG, OriGene, Rockville, MD) or empty vector (pcDNA3.1) as a control mixed with Lipofectamine 2000 (Life Technologies) transfection reagent according to the instructions of the manufacturer. Cells were recovered overnight before any functional studies were performed.

##### Gene Expression and Western Blotting

Total RNA was isolated from transfected cells using the RNeasy mini kit (Qiagen, Toronto, ON, Canada) in accordance with the instructions of the manufacturer. The quality and quantity of RNA were determined by spectrophotometric measurements. Reverse transcription from total RNA and quantitative real-time PCR analysis were performed as described previously (6). Primers were adopted from those used previously (6, 18) or designed using Primer3 software (NCBI) (primer sequences are available upon request). Data were normalized to β actin mRNA. ZIP6- and ZIP7-targeted cDNA plasmid- and empty vector (pcDNA3.1)-treated MIN6 β cells were lysed in radioimmune precipitation assay buffer (Cell Signaling Technology) containing protease and phosphatase inhibitor mixture (Cell Signaling Technology). Lysates were loaded onto a 10% SDS-PAGE gradient gel (Bio-Rad) and transferred onto a PVDF membrane using Trans Blot Turbo (Bio-Rad). The membrane was probed with anti-HA (1:1000, Covance Inc., Montreal, QC, Canada), anti-FLAG (1:1000, Sigma), anti-phospho-ERK (1:1000, Cell Signaling Technology), and anti-α actinin antibodies (1:1000, Cell Signaling Technology), followed by anti-mouse (1:5000) or anti-rabbit (1:5000) secondary antibodies (Cell Signaling Technology), and imaged using Kodak Imager 4000pro (Molecular Imaging Systems, Carestream Health Inc., Rochester, NY) within the linear range of intensity. Coimmunoprecipitation experiments were performed to examine the interaction between proteins. HA-tagged ZIP7 and FLAG-tagged GLP-1R plasmids or FLAG-tagged ZIP6 and His-V5-tagged GLP-1R were cotransfected into MIN6 cells using Lipofectamine 2000 (Life Technologies). Anti-FLAG coimmunoprecipitation was conducted to pull down both bait protein and its interacting partners. 2.5% input and 50% coimmunoprecipitation eluents were loaded for gel electrophoresis and immunoblotting by anti-HA (1:5000, Covance Inc.) or anti-V5 (1:5000, Life Technologies) primary antibody.

##### Confocal Microscopy Imaging

The cellular localization of ZIP6 and ZIP7 was determined in primary dispersed mouse islet cells and MIN6 β cells using confocal microscopy. Staining was performed as described previously (6) with primary anti-HA (1:1000, Covance Inc.), anti-FLAG (1:500, Sigma), anti-KDEL (1:200, Pierce, ThermoFisher), anti-Syntaxin-1a (1:200, Sigma), anti-ZIP7 (1:200, Proteintech, Chicago, IL), anti-ZIP6-Y3 (1:20, an antibody generated in-house by Kathryn M. Taylor, Cardiff University, UK (19)), or anti-insulin (1:100, Dako) primary antibody, followed by Alexa Fluor 488 goat anti-mouse (1:500, Molecular Probes, Life Technologies), Alexa Fluor 555 donkey anti-rabbit (1:500, Molecular Probes, Life Technologies), or donkey anti-guinea pig (1:500, Jackson ImmunoResearch Laboratories) secondary antibodies. Images were acquired on Zeiss confocal microscope at ×40 magnification with an oil lens and analyzed by LSM510 (Zeiss). Colocalization of ZIP6 and ZIP7 with membrane and ER staining was analyzed and determined with Volocity software.

##### Mouse and Human Islet Isolation and Dispersion and GSIS

Mouse islets were isolated by collagenase type V (Sigma) digestion and dispersed by Accutase^TM^ (Millipore) as described previously (20, 21). Human islets from review board-approved healthy donors were provided by the Islet Core and Clinical Islet Laboratory (University of Alberta, Alberta, Canada). GSIS was assessed as described previously (6, 22) using 0, 2, and 20 mm glucose. 48 h after transfection, growth medium was removed, and cells were preincubated with Krebs-Ringer bicarbonate buffer (128.8 mm NaCl, 4.8 mm KCl, 1.2 mm KH_2_PO_4_, 1.2 mm MgSO_4_, 2.5 mm CaCl_2_, 5 mm NaHCO_3_, 10 mm HEPES, and 0.1% BSA (pH 7.4)) for 60 min at 37 °C. Preincubation medium was removed, and cells were incubated under no glucose (0 mm glucose), low-glucose (2 mm glucose), high-glucose (11 or 20 mm glucose) and then KCl (20 mm glucose + 30 mm KCl) or GLP-1 (11 mm glucose + 100 mm GLP-1) conditions for 20 min each at 37 °C. Incubation medium was collected after each condition and stored at −20 °C. Ultrapure water was added to each well and then frozen and thawed to lyse cells. DNA content was determined by spectrophotometric measurement. Insulin concentration in collected media fractions and cell lysates was determined using a homogenous time-resolved fluorescence insulin assay (Cisbio), in accordance with the instructions of the manufacturer, on a PHERAstar plate reader (BMG Labtech, Ortenberg, Germany). Insulin levels were then normalized to DNA content for each treatment.

##### High Content Imaging and Microscopy

Images were acquired and analyzed on a Thermo Fisher Cellomics ArrayScan VTI HCS reader using iDEV^TM^ software. The filter settings for each dye were as follows: excitation/emission, 494/516 nm for FluoZin^TM^3AM and Fluo4AM (Molecular Probes, Life Technologies); excitation/emission, 644/655 nm for CellROX (Molecular Probes, Life Technologies); and excitation/emission, 350/461 nm for Hoechst 33342 (Molecular Probes, Life Technologies). Each dye was loaded into live MIN6 β cells or dispersed mouse islet cells according to the recommendations of the manufacturer. For transmission electron microscopy images, MIN6 cells were transfected with either scrambled siRNA or targeted siRNA for ZIP6 and ZIP7 knockdown and fixed, and images were acquired as described previously (23). Granule number was quantified manually using ImageJ software (24). Total Internal Reflection Fluorescence Microscopy images were acquired with a Nikon TE2000U TIRF microscope at 5 Hz with a 100-ms exposure time. Insulin granule mobilization and exocytosis were analyzed by Matlab (Math Works), ImageJ (National Institutes of Health), and Igor Pro software. For a detailed analysis, refer to a previous publication (25).

##### Mitochondrial Membrane Potential

Primary dispersed mouse islets were transfected with either scrambled siRNA or targeted siRNA for knocking down ZIP6 and ZIP7 expression 48 h prior to loading with rhodamine 123 (25 μg/ml, 10 min) in 2.8 mm glucose imaging buffer. Cells were treated with extra glucose (final concentration, 20 mm) to observe the corresponding change in mitochondrial membrane potential. Images were taken at 10-s intervals at an excitation of 511 nm with an Olympus IX70 inverted epifluorescence microscope in combination with an Ultrapix camera and a computer with PTI imaging software, as described previously (26).

##### Caspase 3/7 Activity Assay

MIN6 cells were seeded onto a 96-well plate and treated. 48-h treatment with 400 μm palmitic acid was used to induce apoptosis as a positive control. Cleaved caspase 3/7 was assayed according to the protocol of the manufacturer (G8091, Promega).

##### xCelligence

The xCelligence system was operated according to the instructions in the user manual (ACEC Biosciences Inc., San Diego, CA). Briefly, MIN6 cells were seeded on to 96-well E-plates, and then cell growth was monitored and recorded as cell index values every 15 min over 50 h.

##### Membrane Yeast Two-hybrid Analysis of GLP-1R in a Human and Mouse Islet cDNA Library

The membrane yeast two-hybrid analysis was performed by Dualsystem Biotech Inc. (Schlieren, Switzerland). The technology and the bait vector pCCW-ste-hGLP-1R-cub have been described previously (27, 28).

##### Statistics

Statistical significance was assessed using Student's *t* test, Welsh *t* test, and one-way or two-way analysis of variance for repeated measures, followed by a Bonferroni post-test comparison where required. *p* < 0.05 was considered significant. All data are presented as mean ± S.E.

## Results

### 

#### 

##### ZIP Family Gene Expression in MIN6 Cells and Human and Mouse Islets

**FIGURE 1.**
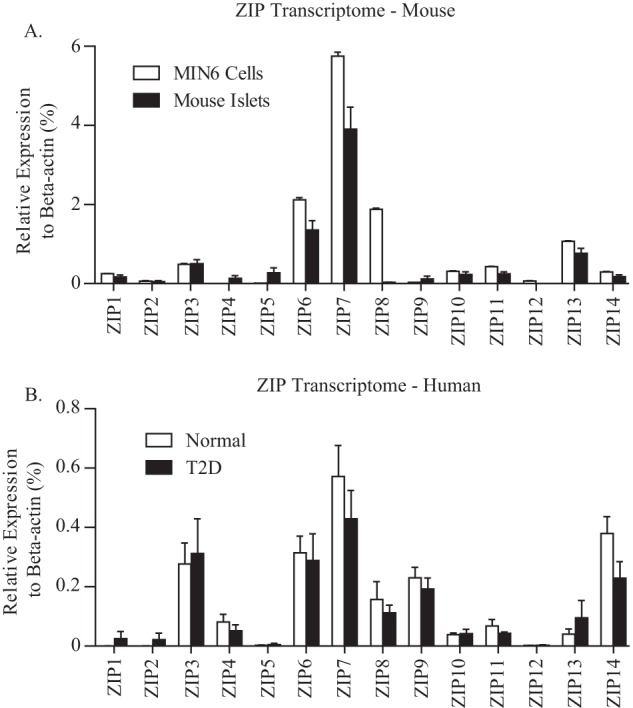
**ZIP family gene expression in MIN6 cells and human and mouse islets.** *A* and *B*, quantitative PCR analysis of the ZIP transcriptome in MIN6 β cells and mouse islets (*n* = 4–6) (*A*) and human islets from normal glucose-tolerant and type 2 diabetic individuals (*n* = 5–13) (*B*). Values were normalized to β-actin mRNA and represented as mean ± S.E.

Several reports have examined the expression of ZIP isoforms in tissues including the GI tract, central and peripheral nervous systems, prostate, liver, kidney, and pancreas (4, 29–33). Here we profile the expression of all 14 ZIP isoforms (Slc39a1–14) in human and mouse pancreatic islets and MIN6 pancreatic β cells. Among the genes examined, ZIP6 and ZIP7 were the most abundantly expressed in both islets and MIN6 cells. We found that the expression level of ZIPs was comparable between MIN6 cells and mouse islets, with the exception of ZIP4, ZIP5, and ZIP8 (Fig. 1*A*). In human islets, we found ZIP3, ZIP8, ZIP9 and ZIP14 expressed at levels comparable with ZIP6 and ZIP7 (Fig. 1*B*). Because zinc plays important roles in pancreatic β cell function, and ZnT8 has been identified as a key risk locus for type 2 diabetes (T2D) in genome-wide association studies, it is reasonable for us to speculate that there may also be a dysregulated ZIP expression profile in islets from diabetic patients. Indeed, we observed a general trend (without statistical significance) of an altered ZIP expression profile in T2D islets compared with those obtained from normal, glucose-tolerant individuals (Fig. 1*B*).

##### Cellular Localization of ZIP6 and ZIP7

**FIGURE 2.**
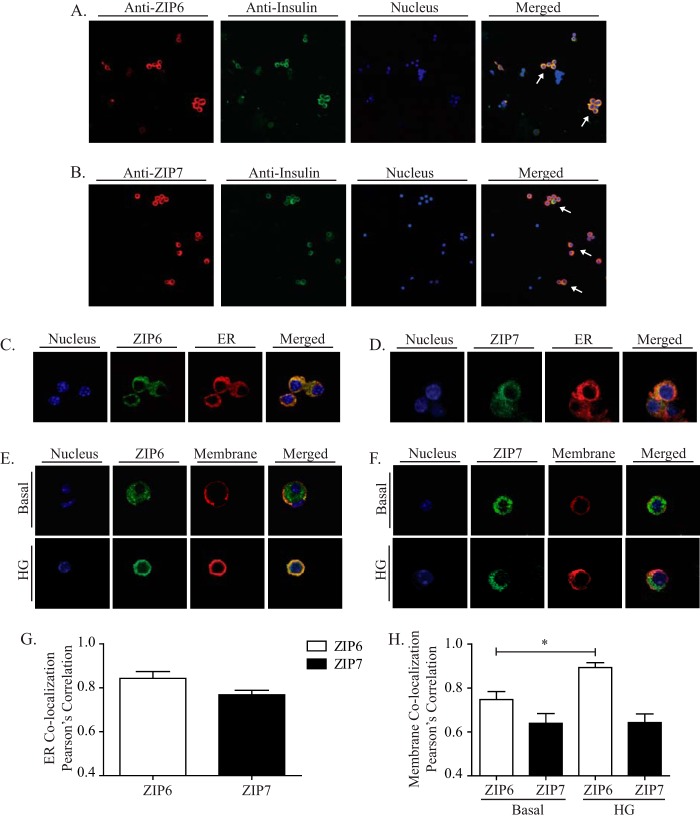
**Cellular Localization of ZIP6 and ZIP7.** *A* and *B*, representative confocal images of endogenously expressed ZIP6 and ZIP7 in dispersed mouse islet cells (*blue*, nucleus; *red*, ZIP6 or ZIP7; *green*, insulin; *arrows*, pancreatic β cells). *C–F*, representative confocal images of endogenously expressed ZIP6 and ZIP7 costained with the ER marker KDEL (*C* and *D*) or the membrane marker syntaxin-1a (*E* and *F*) in the basal or glucose-stimulated state (20 mm glucose, 5 min) (*blue*, nucleus; *red*, membrane or ER; *green*, anti-ZIP6 or anti-ZIP7). *G* and *H*, quantitative analysis by Pearson correlation coefficient shows colocalization between ZIP6 and ZIP7 with the ER (*G*) or plasma membrane (*H*). *n* = 3–4. Values are mean ± S.E. *, *p* < 0.05.*HG*, high glucose.

As noted above, ZIP6 and ZIP7 are the most abundantly expressed ZIP genes in MIN6 cells and human and mouse islets. To study the function of ZIP6 and ZIP7 in pancreatic β cells, we first examined their endogenous expression. By using antibodies specifically detecting ZIP6, ZIP7, and insulin, we demonstrated that, in islet cells, endogenous ZIP6 and ZIP7 are mainly expressed in insulin-producing β cells (Fig. 2, *A* and *B*). The detailed cellular localization of endogenous ZIP6 and ZIP7 was then examined further on dispersed islet cells as outlined below. Studies performed on other cell types have suggested that ZIP6 is primarily localized to the ER under basal states and translocates to the plasma membrane under stimulatory conditions (19, 34), whereas ZIP7 is primarily colocalized to Golgi or ER structures (32, 35–37). Costaining ZIP6 or ZIP7 with either a plasma membrane marker (membrane fusion SNARE protein, Syntaxin-1a) or an ER marker (predominantly ER-expressed protein, KDEL) revealed that both ZIP6 and ZIP7 colocalized predominantly with the ER (Fig. 2, *C* and *D*) in dispersed pancreatic islet cells. Quantitative Pearson's correlation coefficient calculations supported this colocalization between ZIP6 and ZIP7 with the ER (Fig. 2*G*). Interestingly, after glucose stimulation, the majority of ZIP6 protein translocates from the ER to the plasma membrane (Fig. 2*E*), consistent with what has been observed previously in breast cancer cells (19), whereas ZIP7 remains unchanged (Fig. 2*F*). Quantitative Pearson's correlation coefficient calculations also showed a significant increase in the colocalization of ZIP6 and the plasma membrane upon glucose stimulation (Fig. 2*H*).

##### SiRNA-targeted ZIP6 and ZIP7 Knockdown in MIN6 Pancreatic β Cells

**FIGURE 3.**
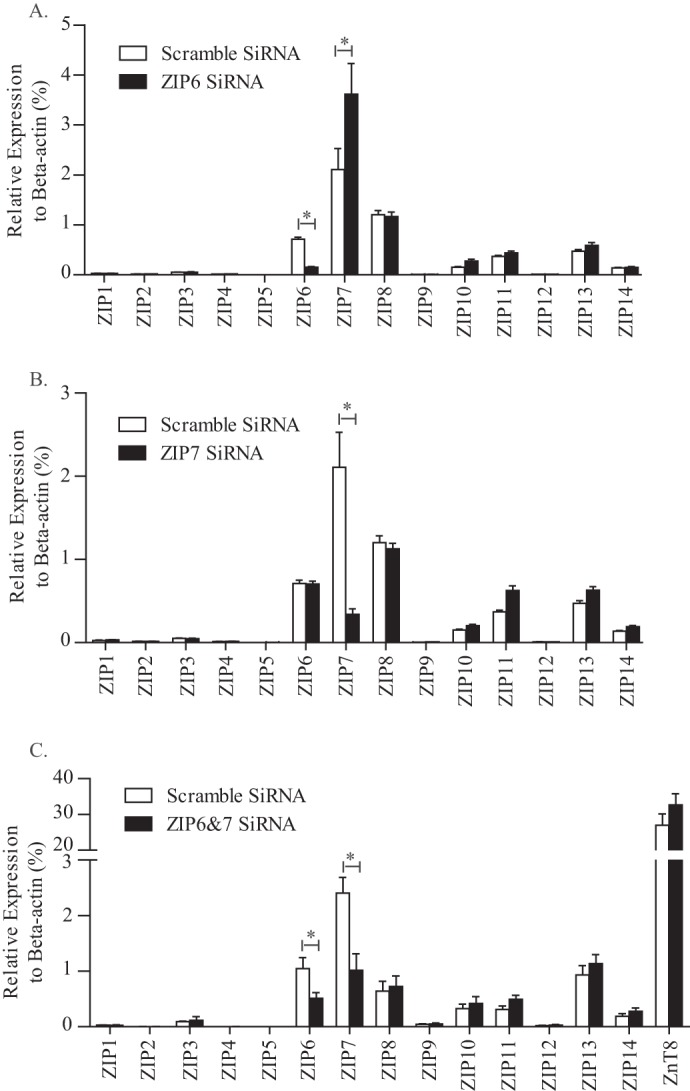
**SiRNA-targeted ZIP6 and ZIP7 knockdown in MIN6 pancreatic β cells.** *A–C*, quantitative PCR analysis of ZIP transcriptome expression upon ZIP6 single knockdown (*A*), ZIP7 single knockdown (*B*), and ZIP6 and ZIP7 double knockdown (*C*). A nonspecific targeted scrambled SiRNA sequence was used as the control. *n* = 4–5. Values were normalized to β-actin are mean ± S.E. *, *p* < 0.05.

To examine the potential role of ZIP6 and ZIP7 *in vitro*, we used targeted siRNA for ZIP6 and ZIP7 knockdown in MIN6 β cells. A knockdown efficiency of 70–80% was achieved for both ZIP6 and ZIP7 (Fig. 3, *A* and *B*). Interestingly, when we knocked down ZIP6, we observed a significant increase in ZIP7 expression (Fig. 3*A*). On the other hand, ZIP7 down-regulation did not cause any change in the expression of other ZIPs (Fig. 3*B*). These findings suggest a possible cooperative role between ZIP6 and ZIP7. Therefore, we used transcriptional silencing of both ZIP6 and ZIP7 in subsequent functional studies. A 50–60% reduction in both ZIP6 and ZIP7 mRNA was achieved, with no significant effect on the expression of other ZIP isoforms or ZnT8 (Fig. 3*C*).

##### Analysis of Cytosolic Zinc Content in MIN6 β Cells and Primary Mouse Islet Cells

**FIGURE 4.**
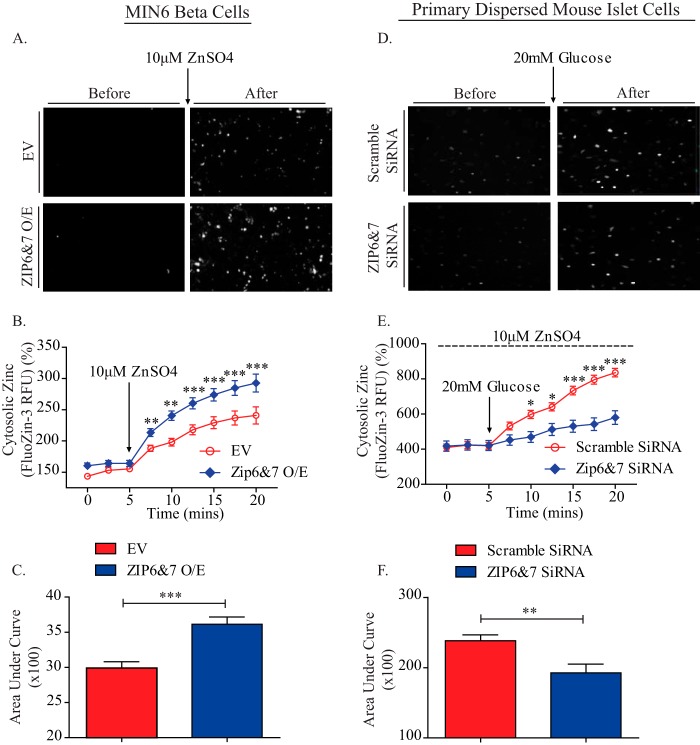
**Analysis of cytosolic zinc content in MIN6 β cells and primary mouse islet cells.** The time course of cytosolic zinc content on cultured live β cells analyzed by the high-throughput, high-content analysis. *A*, representative images taken during the time course of cytosolic zinc content analysis upon ZIP6 and ZIP7 double overexpression. *B* and *C*, quantitative analysis (*B*) and corresponding area under the curve (*C*) of the time course of cytosolic zinc content analysis upon ZIP6 and ZIP7 double overexpression (*O/E*). *D*, representative images taken during the time course of cytosolic zinc content analysis upon ZIP6 and ZIP7 double knockdown. *E* and *F*, quantitative analysis (*E*) and corresponding area under the curve (*F*) of the time course of cytosolic zinc content analysis upon ZIP6 and ZIP7 double knockdown. *n* = 3–4, with 10,000-15,000 individual cells in each experiment. Values are mean ± S.E. *, *p* < 0.05; **, *p* < 0.01; ***, *p* < 0.001. *EV*, empty vector; *RFU*, relative fluorescence units.

To evaluate the role of ZIP6 and ZIP7 in regulating cytosolic zinc influx in live β cells, zinc uptake capacity and concentration were recorded from cells loaded with Fluozin 3AM as a cytosolic zinc indicator. Overexpression of both transporters simultaneously induced a significant increase in zinc uptake upon addition of exogenous ZnSO_4_ (Fig. 4, *A–C*). The significant increase in cytosolic zinc content was sustained in these cells over time (Fig. 4*B*). In complimentary studies, down-regulation of both ZIP6 and ZIP7 expression did not affect basal zinc uptake but significantly reduced glucose-stimulated zinc uptake in dispersed primary mouse islet cells in the presence of ZnSO_4_ (Fig. 4, *D–F*). Previous studies have shown that glucose can activate zinc flux and, therefore, raise cytosolic zinc levels in pancreatic β cells (14, 17, 38). Here we demonstrated an important role for ZIP6 and ZIP7 in this process.

##### Cytosolic Zinc Content Is Essential for GSIS in MIN6 β Cells

**FIGURE 5.**
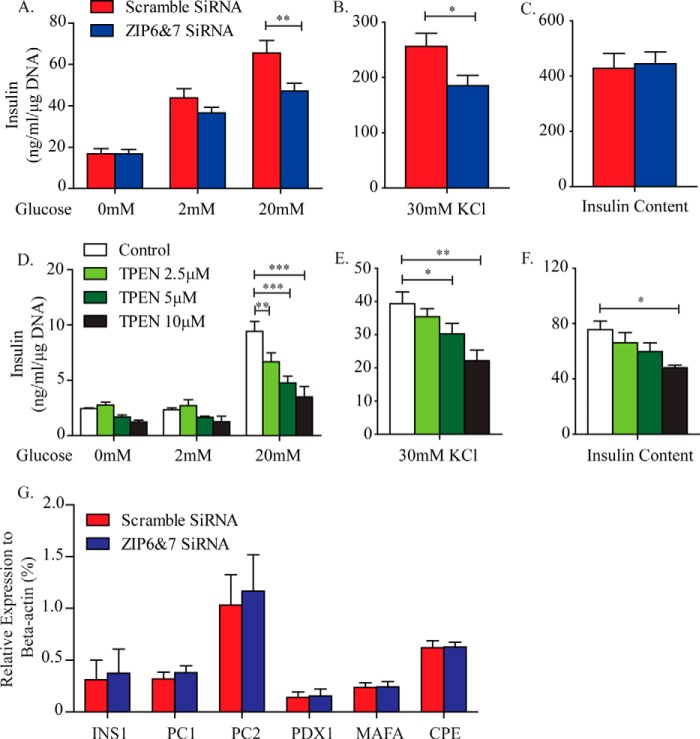
**Cytosolic zinc content is essential for GSIS in MIN6 β cells.** *A*, *B*, *D*, and *E*, glucose- and KCl-stimulated insulin secretion was performed upon ZIP6 and ZIP7 double knockdown (*A* and *B*) and 1 h of TPEN pretreatment (*D* and *E*). *C* and *F*, intracellular insulin content measured upon ZIP6 and ZIP7 double knockdown (*C*) and 1 h TPEN pretreatment (*F*). Insulin levels were normalized to DNA content for each treatment. *G*, quantitative PCR analysis comparing gene expression levels of INS1, PC1, PC2, PDX1, MAFA, and carboxypeptidase E (*CPE*) between scrambled and ZIP6 and ZIP7 siRNA-treated MIN6 cells. A nonspecific scrambled siRNA sequence was used as a control. *n* = 5–6. Values are mean ± S.E. *, *p* < 0.05; **, *p* < 0.01; ***, *p* < 0.001.

As we and others have reported previously, the zinc efflux transporter ZnT8 regulates insulin secretion in part through alterations in insulin secretory granule zinc content (6, 7). We hypothesized that zinc influx transporters may have a role in insulin secretion via alterations in cytosolic and/or organelle-specific zinc pools. Double knockdown of ZIP6 and ZIP7 in MIN6 β cells significantly impaired insulin secretion upon stimulation with high glucose and the secretagogue KCl (Fig. 5, *A* and *B*). Interestingly, the reduction in insulin secretion was not observed in MIN6 cells in which ZIP6 or 7 was knocked down individually (data not shown), further suggesting that a compensatory effect may exist between these ZIP isoforms (Fig. 3, *A* and *B*). The impairment in glucose-stimulated insulin secretion was not due to measurable alterations in insulin biosynthesis because total insulin content was not altered (Fig. 5*C*), nor was the expression of key genes involved in insulin biosynthesis (Fig. 5*G*).

To better delineate whether impaired insulin secretion in ZIP6 and ZIP7 knockdown cells is caused by reduced cellular zinc content, we utilized a zinc chelator, TPEN (39–41), to mimic this condition. TPEN reduced insulin secretion in a dose-dependent manner when stimulated with glucose (Fig. 5*D*) or KCl (Fig. 5*E*). In line with previous studies (42, 43), TPEN dose-dependently affected insulin content, with low concentrations (2.5 and 5 μm) having no effect but a higher concentration (10 μm) decreasing insulin content in MIN6 cells (Fig. 5*F*). Taken together, the data strongly suggest that ZIP6 and ZIP7 are important for maintaining cytosolic zinc content and, therefore, regulating insulin secretion and biosynthesis in pancreatic β cells.

##### Down-regulation of ZIP6 and ZIP7 Expression Induces Oxidative Stress but Not Apoptosis

**FIGURE 6.**
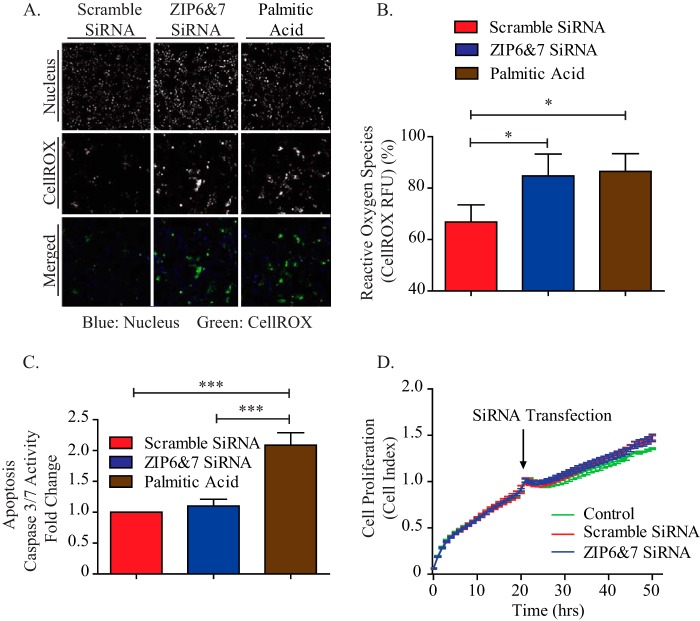
**Down-regulation of ZIP6 and ZIP7 expression induces oxidative stress but not apoptosis.** *A*, representative images taken by the ThermoScientific Cellomics high-throughput machine during ROS measurement upon ZIP6 and ZIP7 knockdown. *B*, quantitative analysis of ROS measurements. *C*, cell death study (cleaved caspase-3/7 activity) upon ZIP6 and ZIP7 knockdown. 400 μm palmitic acid was used as an inducer of oxidative stress and apoptosis. *D*, MIN6 cell growth was monitored continuously by the xCelligence system during the process of cell seeding and siRNA treatment. *n* = 4–5. Values are mean ± S.E. *, *p* < 0.05; ***, *p* < 0.001. *RFU*, relative fluorescence units.

The decreased insulin secretion in ZIP6 and ZIP7 knockdown cells could be caused by impairment of the stimulus-secretion coupling apparatus, exocytotic machinery, and/or through induction of cell toxicity and death. Increasing evidence suggests that zinc content changes can trigger oxidative stress, causing apoptosis (44–46). To examine this, CellROX Deep Red was used to measure a broad range of reactive oxygen species (ROS) in MIN6 β cells. Interestingly, a significant increase in ROS was observed in ZIP6 and ZIP7 knockdown cells (Fig. 6, *A* and *B*). This was similar to a response induced by treatment with palmitic acid, a known inducer of oxidative stress. However, we observed no changes in cleaved caspase 3/7 activity (47, 48) after down-regulation of ZIP6 and ZIP7 expression (Fig. 6*C*), ruling out apoptosis. Concurrently, we also employed the xCELLigence system to record cell proliferation in real time. We observed no significant difference over 50 h during siRNA treatment (Fig. 6*D*). Therefore, impaired GSIS with ZIP6 and ZIP7 knockdown is associated with increased ROS, but not because of cell death.

##### Down-regulation of ZIP6 and ZIP7 Expression Does Not Affect Glucose Metabolism or Calcium Flux

**FIGURE 7.**
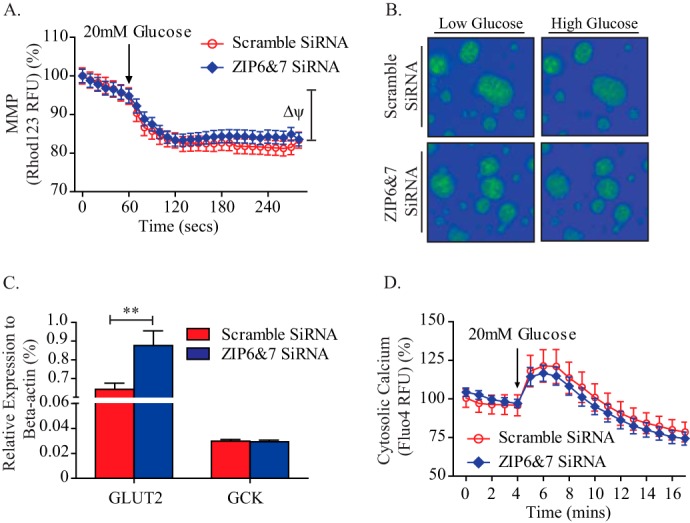
**Down-regulation of ZIP6 and ZIP7 expression does not affect glucose metabolism or calcium signaling.** *A*, glucose-induced hyperpolarization of the MMP was compared between scrambled and ZIP6 and ZIP7 siRNA-treated mouse dispersed islet cells. *B*, representative images from the MMP assay. *C*, quantitative PCR analysis and comparison of the gene expression levels of GLUT2 and GCK between scrambled and ZIP6 and ZIP7 siRNA-treated MIN6 cells. *D*, glucose-induced cytosolic calcium content change examined and analyzed between scrambled and ZIP6 and ZIP7 siRNA-treated MIN6 cells. *n* = 4–5. Values are mean ± S.E. **, *p* < 0.01. *RFU*, relative fluorescence units; *GCK*, glucokinase.

Recent studies have indicated a potential role for ZIP7 in the regulation of glucose metabolism in skeletal muscle cells (49). To determine whether ZIP6 and ZIP7 knockdown impairs glucose metabolism and, therefore, reduces GSIS, we used real-time live imaging (PTI) to record MMP changes upon acute glucose load. In dispersed mouse islet cells, no difference was observed in glucose-stimulated hyperpolarization of the MMP between scrambled and ZIP6 and ZIP7 siRNA-treated cells (Fig. 7, *A* and *B*). In agreement with this result, the expression of key genes involved in glucose uptake (glucose transporter 2, GLUT 2) and metabolism (glucokinase, GCK) were not attenuated with ZIP6 and ZIP7 knockdown (Fig. 7*C*). Many studies have suggested a synergistic role between Zn^2+^ and Ca^2+^ in the regulation of intracellular kinase-activated signaling pathways (50–53). Our laboratory has shown previously that VGCCs act as relatively specific zinc influx transporters (14), suggesting a possible role for zinc in the regulation of cytosolic calcium homeostasis. Glucose-stimulated cytosolic calcium influx is a vital part of the stimulus-secretion coupling pathway and for insulin granule exocytosis in pancreatic β cells (24, 54, 55). Here we examined whether the disruption in cytosolic zinc homeostasis observed in ZIP6 and ZIP7 knockdown MIN6 cells alters cytosolic calcium signaling to impair insulin secretion. Interestingly, no change was observed in glucose-stimulated cytosolic calcium influx upon ZIP6 and ZIP7 knockdown (Fig. 7*D*). Taken together, these results indicate that impairment of GSIS upon ZIP6 and ZIP7 knockdown is neither due to impaired glucose metabolism nor alterations in cytosolic calcium influx.

##### Down-regulation of ZIP6 and ZIP7 Expression Impairs Insulin Exocytosis

**FIGURE 8.**
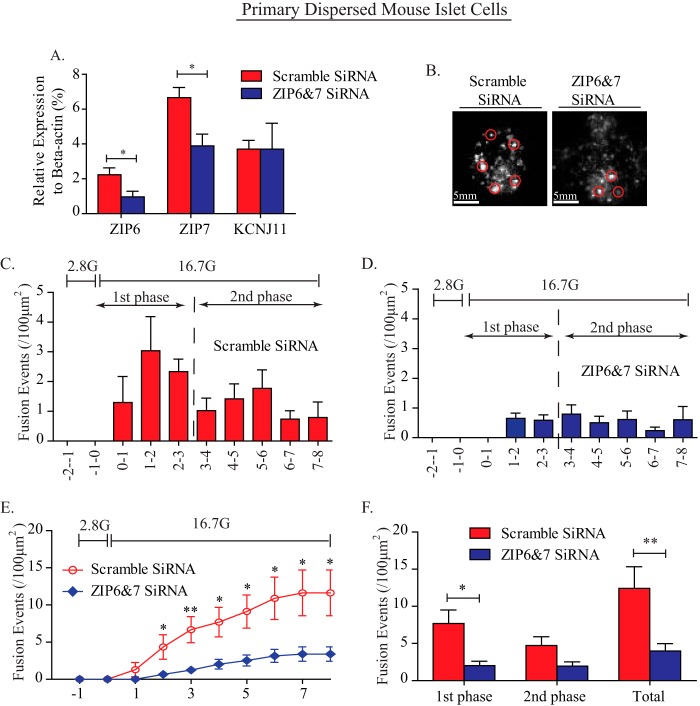
**Down-regulation of ZIP6 and ZIP7 expression impairs insulin exocytosis.** *A*, quantitative PCR analysis of ZIP6 and ZIP7 expression upon double knockdown in dispersed mouse islet cells. A nonspecific scrambled siRNA sequence was used as a control. TIRF measuring was used to measure insulin exocytosis in dispersed mouse islet β cells upon ZIP6 and ZIP7 double knockdown and adenovirus-induced neuropeptide yeast enhanced green gluorescent protein transfection. *B*, representative TIRF image of docked insulin granules (*red circles*) in dispersed mouse islet β cells. *C* and *D*, histograms of fusion events occurring during the first and second phase of glucose-stimulated insulin secretion in scrambled siRNA-treated (*C*) and ZIP6 and ZIP7-targeted siRNA-treated (*D*) mouse islet β cells. *E*, cumulative insulin granule fusion events normalized per cell per 100 μm^2^ during stimulation as indicated. *F*, quantitative summary of total insulin granule fusion events in dispersed mouse islet beta cells. *n* = 6. Values are mean ± S.E. *, *p* < 0.05; **, *p* < 0.01.

The absence of alterations to glucose metabolism and stimulus-secretion coupling in ZIP6 and ZIP7 knockdown cells led us to examine insulin exocytosis. We initially employed transmission electron microscopy to look for differences in granule morphology and distribution. Previously, we and others have demonstrated that altered zinc homeostasis through ZnT8 deletion leads to atypical insulin crystallization within granules, which may alter granule docking and exocytosis (6, 7). Transmission electron microscopy images revealed no differences in the number of dense-core insulin granules in MIN6 cells upon ZIP6 and ZIP7 knockdown (data not shown), indicating that down-regulation of ZIP6 and ZIP7 does not affect insulin crystallization. Next we examined insulin granule secretory dynamics by time-lapse total internal reflection fluorescence (TIRF) microscopy. Here insulin granules in dispersed mouse islet β cells were tagged with neuropeptide yeast enhanced green gluorescent protein by adenovirus transduction, followed by siRNA-mediated ZIP6 and ZIP7 knockdown. As shown previously, ∼50–60% knockdown of ZIP6 and ZIP7 was observed (Fig. 8*A*). A significant reduction in the insulin granule exocytosis rate (fusion events) during the first phase of insulin secretion was observed by TIRF in ZIP6 and ZIP7 knockdown islet cells (Fig. 8, *B–F*). Therefore, in ZIP6 and ZIP7 knockdown β cells, the reduced glucose-stimulated insulin granule docking can explain impaired glucose-stimulated insulin secretion.

##### Effect of ZIP6 and ZIP7 on GLP-1-mediated Signaling

**FIGURE 9.**
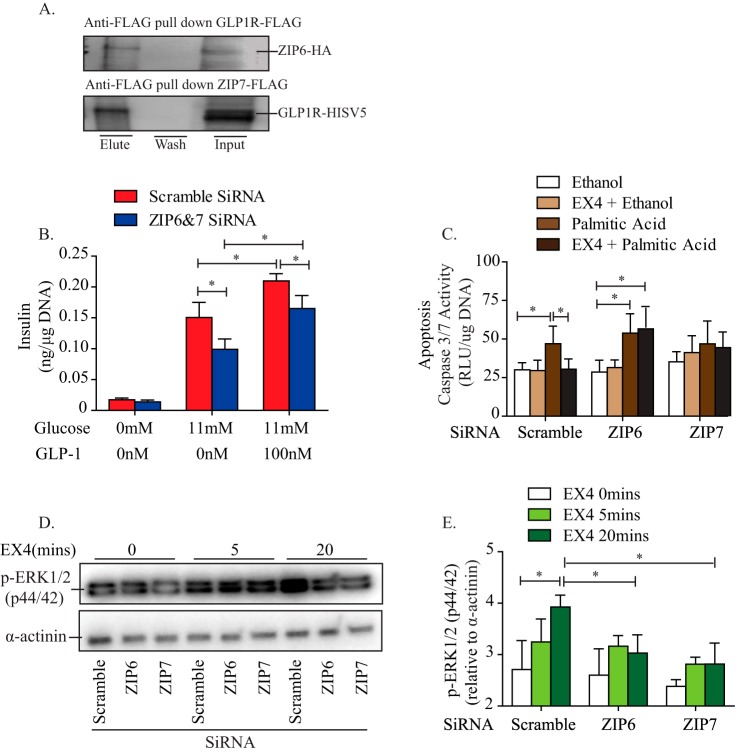
**Effect of ZIP6 and ZIP7 on GLP-1-mediated signaling.** *A*, representative Western blot of coimmunoprecipitation between the GLP-1 receptor with ZIP6 and ZIP7 upon ZIP6 and ZIP7 overexpression, respectively. *B* and *C*, GSIS was performed in INS1 cells (*B*) and cleaved caspase 3/7 activity was measured in MIN6 cells (*C*) with or without Exendin 4 (*EX4*) treatment (100 nm) upon ZIP6 and ZIP7 knockdown. *D* and *E*, representative Western blot (*D*) and quantitative analysis (*E*) of Exendin 4 (100 nm)-induced phosphorylation of ERK1/2 (p44/42). *n* = 3–5. Values are mean ± S.E. *, *p* < 0.05. *RLU*, relative luminescence unit.

GLP-1, acting via the GLP-1 receptor (GLP-1R), has a well established stimulatory effect on glucose-induced insulin secretion from pancreatic islets (56), and it protects rodent β cells from cytokine-induced apoptosis (57). Interestingly, in concurrent studies, ZIP6 and ZIP7 were both identified as putative GLP-1R-interacting proteins in a membrane yeast two-hybrid screen of human and mouse islet cDNA libraries. This method was very similar to what we have reported previously for GLP-1R using a fetal brain cDNA library (28). The interaction between ZIP6/ZIP7 and GLP-1R was validated using coimmunoprecipitation (Fig. 9*A*). Because the interaction between ZIP6 and ZIP7 and GLP-1R indicates a potential functional role for ZIPs in the regulation of GLP-1 signaling, we disrupted the interaction between ZIPs and GLP-1R utilizing siRNA mediated down-regulation of both ZIP6 and ZIP7 expression. Although a reduction in insulin secretion was observed upon GLP-1 treatment, the relative stimulatory effect of GLP-1 on glucose-stimulated insulin secretion remained unchanged compared with the scrambled control (Fig. 9*B*). Therefore, ZIP6 and ZIP7 are not required for GLP-1 augmented glucose-stimulated insulin secretion. To determine whether ZIP6 and ZIP7 are involved in mediating the anti-apoptotic effect of GLP-1, we utilized exendin 4 (EX4, a GLP-1R agonist) and palmitic acid as a proapoptotic stimuli. Interestingly, the down-regulation of ZIP6 but not ZIP7 completely diminished the protective effect of GLP-1 against palmitic acid-induced apoptosis in MIN6 cells (Fig. 9*C*). Correspondingly, down-regulation of ZIP6 expression led to significantly reduced GLP-1-induced p-ERK (Fig. 9, *D* and *E*), one of the key signaling molecules thought to be involved in the anti-apoptotic effect of GLP-1 (58, 59). Together, these findings suggest that ZIP6 is involved in GLP-1R signaling to prevent apoptosis but that it does not directly mediate its effect on insulin secretion.

## Discussion

Because zinc ions are co-secreted together with insulin during insulin exocytosis, β cells require mechanisms to continuously replenish zinc storage by uptake and incorporation into proper intracellular compartments. Despite the well established recognition of the tight relationship between zinc homeostasis and pancreatic β cell function, the participating role of zinc influx transporters has so far not been well examined. Our observation of a possible dysregulated ZIP expression profile in type 2 diabetic islets compared with healthy individuals provides a further rationale to investigate the role of ZIPs in the regulation of pancreatic β cell function. We have demonstrated previously that VGCCs can mediate zinc uptake in pancreatic β cells (14). In this study, we explore the role of two zinc influx transporter members of the ZIP family, ZIP6 and ZIP7, which, as we demonstrate here, are two of the most highly expressed ZIP transcripts in human and mouse islets. Of note, in this study, we observed a difference in the relative abundance of ZIP1 gene expression when compared with our previous work (14), which showed relatively high expression. This may be due to different tissue samples, RNA extraction methods, or the quantitative PCR primer sets employed. However, the relative abundance of ZIP1 compared with other ZIP isoforms shown in this study is strikingly similar to the ZIP expression profiles of our more recent paper (7) and that published by Bellomo *et al.* (17).

The cellular localization of ZIP6 and ZIP7 suggests that these transporters can work in tandem to regulate cytosolic zinc content either by bringing extracellular zinc into cells (60–62) or by pumping ER-stored zinc into the cytosol when needed (35). Importantly, to restore the cellular zinc content after glucose stimulation, ZIP6 appears to be capable of relocating to the plasma membrane from the ER to facilitate zinc influx (Fig. 2, *E* and *H*). This is consistent with previous observations of ZIP6 activation in breast cancer cells (19). Therefore, ZIP6 and ZIP7 likely function to increase cytosolic zinc via increased uptake or reuptake of zinc under basal conditions and in response to glucose to replenish cellular and intragranular zinc during/after insulin secretion.

Interestingly, a significant compensatory increase of ZIP7 expression occurred upon targeted siRNA knockdown of ZIP6, suggesting a tight cooperative relationship between ZIP6 and ZIP7. This led us to use a double knockdown or overexpression approach throughout our study. We performed population studies by using a Cellomics-based high-throughput screening platform where multiple cellular targets and processes occurring in live cells were analyzed over time and space in a non-biased way (44, 63–65). In agreement with previous findings in both dendritic cells (60) and breast cancer cells (37), impaired zinc homeostasis was observed after altered expression of ZIP6 and ZIP7. Notably, because of their subcellular localization, the increase in cytosolic free zinc content we observed here in ZIP6- and ZIP7-overexpressing cells may result not only from enhanced influx of zinc ions from the extracellular space but also from intracellular release from the ER. The compensatory increase in ZIP7 we observed following ZIP6 knockdown suggests that zinc release from internal stores may also be critical to facilitate the process of insulin co-crystallization with zinc and its subsequent exocytosis, emphasizing the requirement for zinc in maintaining proper glucose-stimulated insulin secretion. Indeed, down-regulation of ZIP6 and ZIP7 expression together elicited a significant reduction in the glucose-stimulated increase of cytosolic zinc content in dispersed mouse islet β cells. However, previous studies from our laboratory (14) and a recent paper (17) both suggest that zinc can also enter cells via VGCCs under glucose-stimulated conditions. To dissect the roles of VGCCs and ZIPs in the regulation of cytosolic zinc content, we used the VGCC blocker nifedipine (80 μm) to acutely block the function of VGCCs, as we reported previously (14). In the presence of nifedipine, the knockdown of ZIP6 and ZIP7 in MIN6 β cells was still able to reduce glucose-stimulated cytosolic zinc accumulation (data not shown), suggesting that VGCCs and ZIPs function independently to regulate cytosolic zinc content upon glucose stimulation. Although our data indicate that ZIPs play a major role in regulating β cell zinc content, further experiments are warranted to clearly assess the relative contribution of the VGCC in this process.

Furthermore, knockdown of ZIP6 and ZIP7 disrupted zinc homeostasis and impacted overall β cell function, as demonstrated by significantly impaired glucose- and depolarization (KCl)-stimulated insulin secretion. Again, consistent with a requirement of zinc for normal β cell function, the zinc chelator TPEN caused depletion of cytosolic zinc content, which also lead to an impairment of β cell function. However, under the experimental conditions used here, we observed no significant difference in either intracellular insulin content or expression levels of any genes involved in insulin synthesis/processing upon ZIP6 and ZIP7 knockdown. This suggested that insulin synthesis and processing were not altered. It is possible that decreased β cell zinc accumulation caused by ZIP6 and ZIP7 knockdown may adversely affect zinc accumulation in the insulin granule and, therefore, adversely affect secretion. Indeed, we and others have demonstrated previously that reduced granular zinc in mice lacking the insulin granule-specific zinc transporter ZnT8 is associated with insulin crystallization defects and reduced secretion (7, 12, 13). Although the down-regulation of ZIP6 and ZIP7 did not alter the expression of ZnT8, the impact of reduced cellular zinc content on intragranular zinc concentration is unclear, and further studies are needed to confirm the effect of ZIP6 and ZIP7 knockdown on intracellular compartments of zinc.

Impairment of the ability to respond to the increased demand for insulin secretion is a key element of β cell failure in T2D (66). Here we show a general trend toward the dysregulated expression of ZIPs in islets from T2D patients compared with normal glucose-tolerant individuals. It is therefore reasonable to speculate that reduced expression of key ZIPs, including ZIP6 and ZIP7, may disrupt zinc homeostasis and produce subsequent defects in insulin secretion and reduce β cell viability, potentially increasing the risk of developing diabetes. There are numerous reports supporting a significant interaction between impaired zinc homeostasis and diabetes (67–70), and zinc supplementation has beneficial effects on improving glycemic control in diabetic patients (67, 71, 72). Interestingly, our data suggest that increasing cellular zinc alone is not sufficient to enhance β cell insulin secretion because overexpression of ZIP6- and ZIP7-increased cytosolic zinc has no effect on insulin secretion in healthy β cells. Therefore, zinc does not directly stimulate insulin secretion and must influence β cell function, perhaps protecting against specific forms of β cell dysfunction to maintain insulin secretion.

Our data strongly support the idea that zinc and, by extension, ZIP6 and ZIP7 have a protective role against β cell dysfunction through numerous pathways. One possible role for zinc that is supported by our findings is in the process of granule docking and insulin exocytosis. The actin cytoskeleton is considered the key mediator of biphasic insulin release because it regulates insulin granule docking at the cell periphery (73), and zinc has been shown previously to regulate cytoskeleton dynamics as an important component in stabilizing microtubule structure in neurons (74). In our study, we observed that down-regulation of ZIP6 and ZIP7 resulted in fewer docked granules during both first- and second-phase insulin secretion, indicating a defect in actin cytoskeleton-mediated exocytosis. Interestingly, defects in first-phase insulin secretion are characteristic of T2D, consistent with a possible reduction in ZIP7 expression in diabetic islets. Therefore, we suggest here that there may exist a functional relationship between cytosolic zinc homeostasis and insulin exocytosis mediated by a zinc-facilitated remodeling of the actin cytoskeleton.

Oxidative stress is strongly associated with β cell dysfunction in the development of T2D, and previous studies have shown that maintaining the optimal cellular zinc content is critical for preventing oxidative stress and subsequent cell death in various cell types (75–78). Superoxide dismutases act as one of the major antioxidant enzyme families to protect pancreatic β cells from being damaged by ROS. Zinc is the cofactor of superoxide dismutase and is essential for maintaining proper superoxide dismutase activity (79). Studies have shown that both superoxide dismutase expression and activity are diminished in zinc-deficient cells (80, 81). Indeed, here we show a significant increase in ROS production upon disrupted cytosolic zinc homeostasis because of the down-regulation of ZIP6 and ZIP7 expression, which may also play a partial role in the observed pancreatic β cell dysfunction. Therefore, maintenance of zinc homeostasis through ZIP6 and ZIP7 may play an essential protective role against the development of oxidative stress.

Finally, we made the interesting observation that ZIP6 is an important cellular interactor with the GLP-1 receptor and is required for the protective effect of GLP-1 against palmitic acid-induced β cell apoptosis. Lipotoxicity is thought to be a major underlying cause of β cell death and dysfunction in T2D. Therefore, loss of ZIP6 would make β cells more susceptible to death. Therefore, ZIP6 and ZIP7 may be essential for maintaining β cell function and survival during periods of stress, such as oxidative stress and lipotoxicity. These ZIPs are also likely critical for proper insulin granule exocytosis and appropriate first-phase insulin secretion.

In summary, this study shows that ZIP6 and ZIP7 are novel and important zinc influx transporters in pancreatic β cells. We demonstrated that maintenance of cytosolic zinc homeostasis through these transporters is functionally important for pancreatic β cells to maintain normal insulin exocytosis and, therefore, insulin secretion. Interestingly, we also show that ZIP6 functions as a cellular interactor with the GLP-1 receptor and participates in mediating the effect of GLP-1 to alleviate β cell apoptosis. Finally, our study suggests that these ZIP transporters (ZIP6 and ZIP7) may represent novel targets for drugs that can enhance β cell survival and insulin secretion.

## Footnotes

7 The abbreviations used are:

ZIP: zinc influx transporter
ZnT: zinc efflux transporter
VGCC: voltage-gated calcium channel
GSIS: glucose-stimulated insulin secretion
ER: endoplasmic reticulum
MMP: mitochondrial membrane potential
T2D: type 2 diabetes
ROS: reactive oxygen species
TIRF: total internal reflection fluorescence
TPEN: *N*,*N*,*N*′,*N*′-tetrakis (2-pyridylmethyl) ethylenediamine.
